# Active mechanical cloaking for unsupervised damage resilience in programmable elastic metamaterials

**DOI:** 10.1098/rsta.2023.0360

**Published:** 2024-07-29

**Authors:** D. Kundu, S. Naskar, T. Mukhopadhyay

**Affiliations:** ^1^ Theoretical and Applied Mechanics Program, Northwestern University, Evanston, IL, USA; ^2^ Faculty of Physical Sciences and Engineering, University of Southampton, Southampton, UK

**Keywords:** mechanical cloaking, unsupervised damage resilience, piezoelectric lattice materials, elastic metamaterials, active cloaking, intelligent digital twins

## Abstract

Owing to the architected void-filled low-density configurations, metamaterials are prone to defects during the complex manufacturing process, or damages under operational conditions. Recently mechanical cloaking has been proposed to shield the effect of such disorders in terms of homogenized mechanical responses. The major drawback in these studies are that the damage location should be known *a priori*, and the cloak is designed around that damaged zone before manufacturing. Such postulation does not allow unsupervised damage resilience during the manufacturing and service life of metamaterials by active reconfiguration of the stress field depending on the random and unpredictable evolution of damage. Here, we propose a radically different approach by introducing piezoelectric lattices where the effect of random appearance of any single or multiple disorders and damages with complex shapes, sizes and distributions can be shielded through active multi-physically controlled cloaks by voltage-dependent modulation of the stress fields within the cloaking region. Notably, this can be achieved without breaking periodicity and any additional material in the cloaking region unlike earlier studies concerning mechanical cloaks. The proposed active class of elastic metamaterials will bring a step-change in the on-demand mechanical performance of critically important structural components and unsupervised damage resilience for enhanced durability and sustainability.

This article is part of the theme issue ‘Current developments in elastic and acoustic metamaterials science (Part 1)’.

## 1. Introduction

Elastic mechanical metamaterials have been proven to be promising to develop a range of lightweight multifunctional engineering structures. Mechanical metamaterials are artificially engineered microstructures where the effective properties can be obtained through a conducive nexus among intrinsic material properties, geometry and mechanics, leading to extreme, tunable and multifunctional mechanical behaviour, which are not normally achievable in naturally occurring materials. Over the last decade or so, mechanical metamaterials [1,2] have indubitably demonstrated unprecedented abilities in terms of specific stiffness [3–5], auxeticity [6], impact resistance [7,8], energy absorption [9,10], energy harvesting [11,12], fracture toughness [13,14], variable permeability [15], wave propagation and modulation [16–18], shape transformation [19], critical properties concerning sensors and actuators [20,21] and structural designs with better stress distribution and deformation resistance. This has led to the accelerated adoption of such architected materials in technologically demanding sectors such as aerospace, biomedical, mechanical and civil for achieving extreme or tunable mechanical properties with multi-objective, and often conflicting demands. While elastic metamaterials have emerged to be attractive for developing lightweight engineering structures, owing to their architected void-filled low-density configurations, they are prone to defects during the complex manufacturing process or damage under operational conditions. The central theme of this paper is to propose the concept of active mechanical cloaking to shield the effect of such damages and defects in terms of homogenized mechanical responses.

In general, lattice-based mechanical metamaterials are cellular materials with periodic groupings of unit cells comprising unique tailor-made geometry and patterns to accomplish exceptional bulk properties. Such materials have grown in importance recently as a result of exceptional computational developments in optical, electromagnetic, mechanical and acoustic fields along with the ability to manufacture complex microstructural geometries. These materials are manufactured at the nano-/microscales with complicated physics-informed designs and arrangements of lattice structures, resulting in superior and often unique mechanical properties at the macroscale. To be used as structural components in diverse devices and systems, lattice-based mechanical metamaterials must have their effective elastic characteristics defined. Numerous studies have been reported on various lattice geometries to find and characterize key factors that influence elastic properties. The effective elastic characteristics are controlled by a number of passive variables including geometry, mechanics and the constitutive behaviour of intrinsic materials [22,23]. While a wide range of effective material properties can be achieved in such passive material microstructures, the properties cannot be tuned in an on-demand basis after they are manufactured. The recent developments in this direction include bi-level topology architected optimum metamaterials [5], hierarchical metamaterials [24], disordered metamaterials [25], anti-curvature metamaterials with programmed curvature [26–29], multi-material and space-filled lattices [30,31], origami- and kirigami-inspired metamaterials [19,32], to mention a few. Recently, the concept of pneumatic elastostatics and deployability in mechanical metamaterials has been proposed based on inflatable lattices that can exhibit extreme specific stiffness along with on-demand tunability [33].

Multi-physical mechanics involving external stimuli like electrical and magnetic fields, light, temperature and shape memory effects have recently been exploited to achieve on-demand programmability in lattice metamaterials [1]. For example, the effective elastic moduli can be actively controlled in piezoelectric [34–36] lattices as a function of voltage, leading to modulation of stiffer or softer behaviour of a single lattice architecture in an on-demand framework as per operational demands, even after it is manufactured [37,38]. A magnetic field can lead to such on-demand modulation of effective elastic properties (including sign reversal), but in a contactless framework [39,40]. In the present work, we will introduce piezoelectric multi-physical lattices for developing a framework for active cloaking mechanical metamaterials, as discussed in the following paragraphs.

Followed by the fascinating development of invisibility cloak [41], multiple studies have been reported in the field of wave propagation concerning electromagnetics [42], fluids [43], acoustics [44] and quantum matter [45] to develop cloaks. To cloak, an object or a phenomenon occurring in a system is supposed to hide from the path of general perception [46]. Previous literature also shows elastodynamic [47] and hyperelastic cloaking based on prestress [48–50]. One of the most recent trends concerning cloaking in metamaterials is the development of mechanical cloaks dealing with primarily stress and strain fields to hide defects, damages or other adverse features. To identify microstructures displaying such an altered (hidden) distribution in mechanical cloaks, it has usually been pursued by applying coordinate transformation [51] to a given material–parameter distribution and then solving an inverse problem. Mechanical cloaks are often created to alter the elastic response around objects in order to blend them into their standardized surroundings. Some recent works have developed the idea of cloaking the mechanical properties using metamaterials under a thermal gradient [52], geometry transformation adding thickened cell struts [51], lattice transformation [53] and also using optimized material addition [54] in the cloaking region. A recent study through data-driven design approaches proposes adaptable construction of mechanical cloaks with diverse boundary and loading conditions along with various void shapes and numbers [55].

In the above-mentioned works concerning mechanical cloaks, the cloaking is proposed through supervised and passive frameworks, which may cost the structure its life owing to time constraints in developing the cloaks, or alter some of the critical features of the overall structure owing to the addition of the cloaking region after manufacturing. Furthermore, most of the reported works lack practicality in terms of generic damage shapes. The major drawback in such studies on mechanical metamaterials is that the damage location should be known *a priori*, and the cloak is designed around that damaged zone before manufacturing. Such postulation does not allow unsupervised damage resilience during the service life of the metamaterials by active reconfiguration of the stress field depending on the random and unpredictable evolution of damage. Though a recent study has proposed magnetoactive mechanical metamaterial for tunable elastic cloaking [56], the central limitation of knowing a fixed location of damage *a priori* persists. Here, we aim to propose a radically different approach by introducing piezoelectric lattices where the effect of the random appearance of any damage can be shielded through active multi-physically controlled cloaks by voltage-dependent modulation of the stress fields within the cloaking region. According to the operational conditions of different structures and mechanical systems including a range of boundary conditions, single or multiple such disorders and damages with complex shapes and sizes can effectively be kept hidden, ensuring high reliability in the homogenized mechanical behaviour.

**Figure 1.**
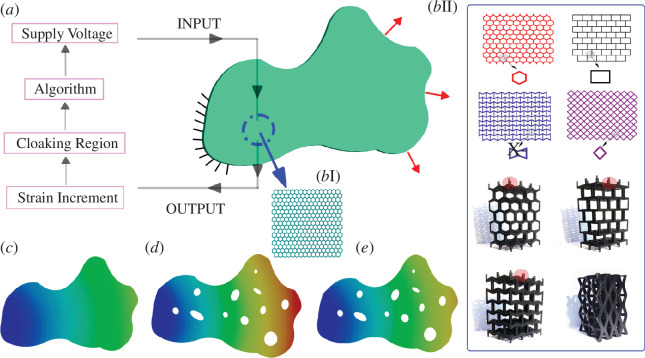
Active mechanical cloaking in elastic metamaterials. (*a*) An arbitrary structural domain with Dirichlet and Neumann boundary conditions, i.e. fixity conditions and loading. Strain measurements are sent as an output to an external circuit board, which decides the cloaking region and the inbuilt algorithm decides the supply voltage to be sent as an input to the structure in an unsupervised framework. (*b*) (I) Lattice-based hexagonal microstructure where each of the beam-like elements is electro-active. (II) Typical representation of other prospective two-dimensional and three-dimensional lattices where the proposed active cloaking can be implemented. (*c*) Undamaged lattice structure with original displacement gradient (minimum ‘blue’ to maximum ‘red’). (*d*) Damaged lattice structure with the corresponding displacement gradient. (*e*) Voltage-dependent modified displacement gradient of the damaged structure after cloaking. The displacement field presented in subfigure (*e*) beyond the cloaking region is supposed to be close to that presented in subfigure (*c*).

**Figure 2.**
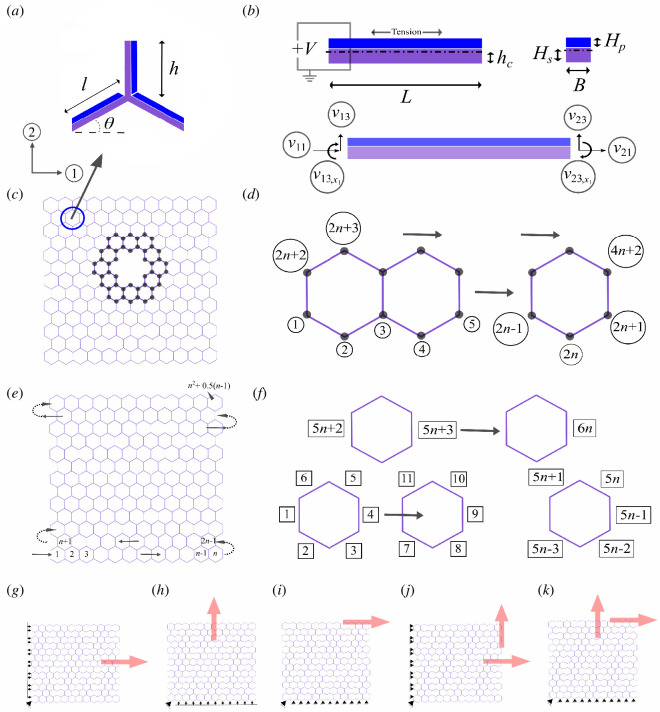
Lattice metamaterials with piezoembedded beam-like elements and lattice-level mechanics for displacement fields including the effect of cloaking. (*a*) The typical unit cell of a hexagonal lattice constructed with composite beams with a substrate material (in violet) bonded to a piezoelectric patch (in blue), representing an unimorph configuration. (*b*) Enlarged representation of a piezoembedded composite beam with the cross-sectional view shown on the right. The basic circuit diagram is also shown along with local degrees of freedom (axial, transverse and rotation). (*c*) A typical representation of a damaged lattice formed with the hexagonal unit cells is shown in subfigure (*a*). The bold members represent the piezoelectric active beams (constituting the cloaking region) and the piezoelectric degrees of freedom are marked. (*d*) The row-wise global node numbering is shown, numbering the odd rows only to include all nodes in the lattice. (*e*) An undamaged lattice formed with the hexagonal unit cells shown in subfigure (*a*). The cell-wise marking is shown using the arrows that help in the development of the finite element connectivity matrix. (*f*) The row-wise global element numbering is shown separately for odd and even rows. (*g–i*) Uni-modal loading considering far-field axial stress along directions 1 and 2, and shear stress along direction 12. (*j, k*) Mixed-mode loading under complex far-field stresses. The subfigures are represented using the general boundary conditions.

For developing active mechanical cloaks, we propose piezoelectric lattice metamaterials (refer to figures 1 and 2*a*) where element-wise (i.e. beam-like elements, shown as sides of the hexagonal units) voltage modulation is conceptualized to control the stress or strain fields in the cloaking regions. It is to be noted that in figure 2*a*, the tri-membered unit cell is just shown to give a better view to the reader of the piezoelectric layer added to the substrate beam. The tessellation of a single unit cell helps in generating the overall lattice. Also, predicting the mechanical response from a unit cell helps understand the effective physical behaviour of undamaged lattices in an efficient computational framework because of the periodicity concept involved here [1,22]. However, in the current paper, we have not used the periodicity concept of the lattice to generate our numerical results as the damaged lattice no longer remains an exact periodic system. Instead, we have used a finite element-based scheme with optimization of the displacement norm for the full lattice system to solve the problem of unsupervised damage resilience. To demonstrate the concepts, we would primarily focus on two-dimensional hexagonal metamaterials with different shapes of randomly placed damages having varying numbers. However, the concept of active mechanical cloaks would be generic in nature and it can be implemented in other two-dimensional and three-dimensional lattice metamaterials (refer to figure 2*b*). Hereafter, we discuss the concept of active cloaking in elastic metamaterials in §2, followed by the underlying mathematical formulations and computational framework in §3. Numerical demonstrations of active cloaking are furnished in §4 considering different shapes, numbers and randomized locations of damages along with various conditions of far-field stresses. Finally, §5 provides concluding remarks and perspective.

## 2. Concept of active cloaking and damage resilience

Mechanical cloaks are material systems that are designed to alter the elastic response around defects/voids to make them unrecognizable from their homogeneous surroundings. To cloak a defect implies making it invisible in terms of a physically accessible field (such as strain). Cloaking effects have shown notable progress in the design of lattice metamaterial for electromagnetic waves around a hidden object. Optical, thermal and electric cloaks are generally designed using approaches based on material–parameter transformations [55]. Unfortunately, as continuum equations are not form-invariant under generalized coordinate transformations, these cloak design methodologies cannot be used to design mechanical cloaks. As per the current state of knowledge, it is not possible to cloak damages or defects in metamaterials under operational conditions as they appear in real-time after manufacturing. As a result, existing mechanical cloak methodologies have been restricted to small pre-defined voids (or defects) with simple shapes and limited boundary conditions.

In this paper, for on-demand cloaking, active materials will be exploited so that the local-level mechanical constitutive laws around the damages can be modulated as a function of external stimuli such as the electric field. The theory of cloaking will be developed based on a computational framework of multi-physical finite element simulations along with numerical optimization which will include the determination of element-wise voltages in the cloaking region to negate the effect of damage in the strain field beyond this region. By re-distributing stress and strain components in an active and on-demand framework based on the location, nature, size and number of damages (either manufacturing or operational in nature), mechanical cloaking as proposed here will facilitate hiding essential voids and cut-outs in structural designs, or unintentional defects which are often the sources of deviation from intended structural performance and further failure initiation (damage resilience). It will significantly enhance the durability and lifetime of a structure without compromising on mechanical performances like strength and stiffness. Furthermore, since the current framework relies on external multi-physical stimuli like voltage in the cloaking region for altering the stress and strain fields, it is not necessary to reinforce the cloaking region through optimally introduced additional elements in the lattice, as commonly followed in the mechanical cloaks proposed in metamaterials. From a sustainability viewpoint, mechanical cloaking without any additional weight would be crucial for a range of engineering applications. Since the periodicity in lattice geometry is not broken in the present framework, it will also be helpful for manufacturing and scalability. The realization of active cloaking will bring a step-change in the field of mechanical cloaking since the location and intensity of damage (/disorder) is not necessarily needed to be known *a priori* before manufacturing. According to the service life conditions of different structures and mechanical systems including a range of boundary conditions, single or multiple such disorders with complex shapes and sizes can effectively be kept hidden.

To elucidate the concept of active mechanical cloaking, we consider an arbitrary domain of a lattice metamaterial. As demonstrated schematically in figure 1, the arbitrary domain is subjected to certain loading and boundary conditions, resulting in a stress or strain field as shown in subfigure (*c*) when there is no defect or damage. If some defect evolves in the same domain under the same loading and boundary conditions, the stress or strain field would change, as depicted in subfigure (*d*). However, we propose to design the domain in such a way that the stress or strain field will remain unaltered beyond certain boundaries around the defects through element-wise (implying each beam-like element of the piezoelectric lattice metamaterial) application of external stimuli in the cloaking region. The objective here is to minimize the discrepancies between the stress or strain fields of subfigure (*c*, *d*) beyond the cloaking region through the application of optimized voltages in each beam-like element within the cloaking region. Note that the applied voltage would be optimum in the current framework since we propose to apply voltages element-wise selectively and individually depending on the respective demands to negate the effect of altered stress or strain fields owing to the introduction of defects. Further, unlike the current state of the art, we would be able to achieve active cloaking without breaking geometric periodicity in the cloaking region, or without having additional material therein.

In the current study, our key focus is to negate the effect of damage in edge deformation of the damaged lattice and converge to the displacement or strain field of the undamaged lattice beyond the cloaking region. This objective is similar to other studies discussed in the preceding paragraphs concerning mechanical cloaks, but overcoming a range of critical limitations as pointed out. We have achieved the stated objective in an on-demand framework by proposing element-wise voltage optimization within the cloaking region at two levels concerning the alteration of stress and strain fields through external stimuli. In the first stage, we obtain a voltage matrix for on-demand activation of the cloaking region. Subsequently, we introduce an automated voltage augmentation factor to accentuate the effect of electrical stimuli for negating the influence of damage in terms of the displacement or strain fields near the edges. This seamless bi-level deformation negation algorithm allows us to save time in the overall iterative simulation process. By limiting the activation to the beams only in the cloaking region, we take a step towards using minimal external electrical energy.

The elastic metamaterial we put forth here will be intelligent in nature to ask the energy array to supply different voltages for activating the target lattice members within the cloaking region. Strain gauges connected to a material or structural domain will be able to detect damage by studying unusual strain values compared with undamaged structures as they evolve. Based on the damaged region, the cloaking region is decided where optimally identified electrical energy is introduced. The algorithm we develop here will determine the external voltage required to mitigate the effect of damage by reducing the discrepancies in global strain fields beyond the cloaking region. It is obvious that the global deflections increase within the structural nodes when it has encountered damage owing to a reduction of stiffness. In the current work, we have proposed to exploit the idea of the converse piezoelectric effect for cloaking damaged honeycomb lattices to tone with the original deformation field of the undamaged lattice.

## 3. Computational framework for demonstrating active and on-demand cloaking

The computational framework for active cloaking in mechanical metamaterials involves a seamless integration of three components: (i) piezoelectric beam elements and their voltage-dependent deformation mechanics, (ii) lattice-level analysis to obtain the strain field under a given loading and boundary conditions, and (iii) multi-objective discrepancy minimization algorithm between the strain fields of damaged and undamaged lattices. A concise flowchart in algorithmic form is presented in algorithm 1. In this study, we have considered different damage scenarios with axis symmetry, bi-axis symmetry, and non-symmetry shapes over the lattice. To check the validity of the proposed framework, we have expanded it further to randomly distributed damage cases by varying the damage positions. We would begin by considering uni-modal axial and shear loading individually, and extend our work to mixed-mode loading with different boundary conditions.

### (a) Mechanics of unimorph piezoelectric beam elements

The mathematical formulation has been presented here showing the development of a piezoelectric stiffness matrix of a composite beam with a substrate and piezoelectric layer having unimorph configuration (refer to figure 2*b*). The vectors, tensors and matrices are written in bold case to avoid confusion. Subscripts and superscripts with s and p , in general, will represent the substrate and piezoelectric layer of the beam, respectively. T in the superscript will indicate the transcript. Considering a beam having the local axis system as $x_{1}-x_{2}-x_{3}$ , the displacements of any arbitrary axis of the beam along the axial and transverse directions at any time, t can be shown using equations (3.1) and (3.2). Because of the bending of the beam, the axial deflection will have components of the axial deformation of the neutral axis, i.e. $v_{1}^{c}(x_{1},t)$ as well as the transverse deformation, $v_{3}^{c}(x_{1},t)$ at the considered section.


(3.1) $$v_{1}=v_{1}^{c}\left(x_{1},t\right)-x_{3}v_{3}^{c}{,}_{x_{1}}\left(x_{1},t\right),$$

(3.2) $$v_{3}\left(x_{1},t\right)=v_{3}^{c}\left(x_{1},t\right).$$

  The total potential energy of the beam with an overall volume of $\Omega_{s}+\Omega_{p}$ can be represented using equation (3.3). It will have the energy components both from the substrate layer as well as the layer comprising the piezoelectric patch, and integration is done separately over the volume. We have represented the engineering strain tensor using ϵ and for the engineering stress tensor, we have used, $\sigma_{p}$ or $\sigma_{s}$ , depending upon the substrate or piezoelectric layer.


(3.3) $$T_{P}=\frac{1}{2}\left(\int_{\;\;\Omega_{s}}\epsilon^{\text{T}}\sigma_{s}d\Omega_{s}+\int_{\;\;\Omega_{p}}\epsilon^{\text{T}}\sigma_{p}d\Omega_{p}\right).$$

Considering the Young’s modulus of the substrate as $E_{s}$ , we can calculate the overall strain, $\epsilon_{11}(x_{1},x_{3},t)$ of the beam and stress component, $\sigma_{11}^{c}(x_{1},x_{3},t)$ of the substrate layer along the direction $x_{1}$ as shown in equations (3.4) and (3.5).


(3.4) $$\epsilon_{11}\left(x_{1},x_{3},t\right)=\frac{\partial v_{1}^{c}\left(x_{1},t\right)}{\partial x_{1}}-x_{3}\frac{\partial^{2}v_{3}^{c}\left(x_{1},t\right)}{\partial x_{1}^{2}},$$

(3.5) $$\sigma_{11}^{s}\left(x_{1},x_{3},t\right)=E_{s}\epsilon_{11}\left(x_{1},x_{3},t\right).$$

Using the same strain component, $\epsilon_{11}(x_{1},x_{3},t)$ , we also calculate the stress component, $\sigma_{11}^{p}(x_{1},x_{3},t)$ of the piezoelectric layer as shown in equation (3.6). Stress in the piezoelectric layer will also depend on the time-dependent electric field $E_{f}(t)$ , i.e. $V/H_{p}$ , the ratio of voltage, V with the piezoelectric layer thickness, $H_{p}$ . Here, ${\bar{p}}_{31}$ is the effective piezoelectric stress constant.


(3.6) $$\sigma_{11}^{p}\left(x_{1},x_{3},t\right)=E_{p}\epsilon_{11}\left(x_{1},x_{3},t\right)+{\bar{p}}_{31}\frac{V}{H_{p}}.$$

The set of equation (3.7a–c) describes the relationship of permittivity constants at constant stress and strain, $p_{33}^{\sigma }$ and $p_{33}^{\epsilon }$ with the elastic compliance at the constant electric field, $c_{11}^{E_{f}}$ and $p_{31}^{k}$ , the piezoelectric constant. The elastic compliance at a constant electric field is the inverse of Young’s modulus, $E_{p}$ of the piezoelectric layer. Finally, we relate ${\bar{p}}_{31}$ , the effective piezoelectric stress constant with $c_{11}^{E_{f}}$ , the elastic compliance at a constant electric field and $p_{31}^{k}$ , the piezoelectric constant.


(3.7*a*) $$p_{33}^{\epsilon }=p_{33}^{\sigma }-\frac{p_{31}^{k^{2}}}{c_{11}^{E}},$$

(3.7*b*) $$E_{p}=\frac{1}{c_{11}^{E_{f}}},$$

(3.7*c*) $${\bar{p}}_{31}=\frac{p_{31}^{k}}{c_{11}^{E_{f}}}.$$

Substituting the above deductions in equation (3.3), for the total potential energy, we get equation (3.8).


(3.8) $$\begin{array}{rl} T_{P}= & \frac{1}{2}\int_{\;\;0}^{L}\left(E_{s}\left(A_{s}{\left(\frac{\partial v_{1}^{c}(x_{1},t)}{\partial x_{1}}\right)}^{2}+I_{s}^{2}{\left(\frac{\partial^{2}v_{3}^{c}(x_{1},t)}{\partial {x_{1}}^{2}}\right)}^{2}-2I_{s}^{1}\left(\frac{\partial v_{1}^{c}(x_{1},t)}{\partial x_{1}}\frac{\partial^{2}v_{3}^{c}(x_{1},t)}{\partial {x_{1}}^{2}}\right)\right)\right)dx_{1}\\ & +\frac{1}{2}\int_{\;\;0}^{L}\left(E_{p}\left(A_{p}{\left(\frac{\partial v_{1}^{c}(x_{1},t)}{\partial x_{1}}\right)}^{2}+I_{p}^{2}{\left(\frac{\partial^{2}v_{3}^{c}(x_{1},t)}{\partial {x_{1}}^{2}}\right)}^{2}-2I_{p}^{1}\left(\frac{\partial v_{1}^{c}(x_{1},t)}{\partial x_{1}}\frac{\partial^{2}v_{3}^{c}(x_{1},t)}{\partial {x_{1}}^{2}}\right)\right)\right)dx_{1}\\ & +\int_{\;\;0}^{L}\left(C_{1}v\frac{\partial v_{1}^{c}(x_{1},t)}{\partial x_{1}}-C_{2}v\frac{\partial^{2}v_{3}^{c}(x_{1},t)}{\partial {x_{1}}^{2}}\right)dx_{1} \end{array}.$$

  The neutral axis of the composite beam constituting the substrate material and the piezoelectric patch can be given as $h_{c}$ from the bottom of the beam using equation (3.9) (refer to figure 2*b*).


(3.9) $$h_{c}=\frac{A_{s}0.5H_{s}+\frac{E_{p}}{E_{s}}A_{p}\left(0.5H_{p}+H_{s}\right)}{A_{s}+\frac{E_{p}}{E_{s}}A_{p}}.$$

Here A , $I^{1}$ and $I^{2}$ are the cross-sectional area, first and second moment of inertia separately shown for the substrate and the piezoelectric layer with subscripts s and p . The equations for these geometric properties are shown in the set of equation (3.10a–c) and (3.11a–c) for the substrate and piezoelectric layers of the beam.


(3.10*a*) $$A_{s}=\int \int_{\;\;\Omega_{s}}dx_{3}dx_{2}=\int_{\;\;0}^{B_{s}}\int_{\;\;\;-h_{c}}^{\;H_{s}-h_{c}}dx_{3}dx_{2}=B_{s}H_{s},$$

(3.10*b*) $$I_{s}^{1}=\int \int_{\;\;\Omega_{s}}x_{3}dx_{3}dx_{2}=\int_{\;\;0}^{B_{s}}\int_{\;\;\;-h_{c}}^{\;H_{s}-h_{c}}x_{3}dx_{3}dx_{2}=\frac{B_{s}}{2}\left(H_{s}^{2}-2H_{s}h_{c}\right),$$

(3.10*c*) $$I_{p}^{2}=\int \int_{\;\;\Omega_{s}}x_{3}^{2}dx_{3}dx_{2}=\int_{\;\;0}^{B_{s}}\int_{\;\;\;-h_{c}}^{\;H_{s}-h_{c}}x_{3}^{2}dx_{3}dx_{2}=\frac{B_{s}}{3}\left(H_{s}^{3}+3H_{s}h_{c}^{2}-3H_{s}^{2}h_{c}\right),$$

(3.11*a*) $$A_{p}=\int \int_{\;\;\Omega_{p}}dx_{3}dx_{2}=\int_{\;\;0}^{B_{p}}\int_{\;\;\;H_{s}-h_{c}}^{\;H_{s}-h_{c}+H_{p}}dx_{3}dx_{2}=B_{p}H_{p}\;,$$

(3.11*b*) $$I_{p}^{1}=\int \int_{\;\;\Omega_{p}}x_{3}dx_{3}dx_{2}=\int_{\;\;0}^{B_{p}}\int_{\;\;\;H_{s}-h_{c}}^{\;H_{s}-h_{c}+H_{p}}x_{3}dx_{3}dx_{2}=\frac{B_{p}}{2}\left(2H_{p}H_{s}+H_{p}^{2}-2H_{p}h_{c}\right),$$

(3.11*c*) $$I_{s}^{2}=\int \int_{\;\;\Omega_{p}}x_{3}^{2}dx_{3}dx_{2}=\int_{\;\;0}^{B_{s}}\int_{\;\;\;H_{s}-h_{c}}^{\;H_{s}-h_{c}+H_{p}}x_{3}^{2}dx_{3}dx_{2}=\frac{B_{s}}{3}\left(H_{p}^{3}+3H_{p}\left(H_{s}^{2}+H_{s}H_{p}+h_{c}^{2}-2H_{s}h_{c}\right)\right).$$

The coupling terms, $C_{1}$ and $C_{2}$ , are shown in equations (3.12*a*,*b*). Note that these expressions are obtained from equations (3.3) and (3.8), wherein voltage-extension and voltage-curvature coupling and their contribution to the total potential energy can be established.


(3.12*a*) $$C_{1}=\int \int_{\;\;\Omega_{p}}\frac{{\bar{p}}_{31}}{H_{p}}dx_{3}dx_{2}=\int_{\;\;0}^{B_{p}}\int_{\;\;\;H_{s}-h_{c}}^{\;H_{s}-h_{c}+H_{p}}\frac{{\bar{p}}_{31}}{H_{p}}dx_{3}dx_{2}=B_{p}{\bar{p}}_{31},$$

(3.12*b*) $$C_{2}=\int \int_{\;\;\Omega_{p}}\frac{{\bar{p}}_{31}}{H_{p}}x_{3}dx_{3}dx_{2}=\int_{\;\;0}^{B_{s}}\int_{\;\;\;H_{s}-h_{c}}^{\;H_{s}-h_{c}+H_{p}}\frac{{\bar{p}}_{31}}{H_{p}}x_{3}dx_{3}dx_{2}=\frac{B_{p}{\bar{p}}_{31}}{2}\left(2H_{s}+H_{p}-2h_{c}\right).$$

  Now, we can write the internal energy in the piezoelectric layer given by *U*_p_, as shown in equation (3.13). The electric field vector can be represented as $E_{f}$ , whereas the vector constituting the electric displacement terms is $v_{d}$ .


(3.13) $$U_{p}=\frac{1}{2}\int_{\;\;\Omega_{p}}E_{f}^{\text{T}}v_{d}d\Omega_{p}=-\frac{1}{2}\left(\int_{\;\;0}^{L}\left(C_{1}V\frac{\partial v_{1}^{c}\left(x_{1},t\right)}{\partial x_{1}}-C_{2}V\frac{\partial^{2}v_{3}^{c}\left(x_{1},t\right)}{\partial x_{1}^{2}}\right)dx_{1}+p_{33}^{\epsilon }\frac{A_{p}}{H_{p}}V^{2}\right).$$

Considering a beam element with two nodes, it will have a total of 6 degrees of freedom, 3 at each node, i.e. axial, transverse and rotation. We represent them using the vector, $v_{p}$ with the components as $v_{ij}$ where i stands for the node and j stands for the direction of deformation considered. Except for the third and sixth components that represent the rotations with first-order differential terms with respect to $x_{1}$ , the other components represent the deformations.


(3.14) $$v_{p}^{\text{T}}=\left[\begin{array}{cccccc} v_{11} & v_{13} & v_{13_{,x_{1}}} & v_{21} & v_{23} & v_{23_{,x_{1}}} \end{array}\right].$$

  The deformation of the neutral axis can be represented as follows considering a shape function matrix, F . The elements of the matrix are represented as $F_{kl}$ where k represents the node and l stands for the loading direction, either axial, transverse or rotation.


(3.15) $$\left[\begin{array}{c} v_{1}^{c}\\ v_{3}^{c} \end{array}\right]=\left[\begin{array}{cccccc} F_{1a} & 0 & 0 & F_{2a} & 0 & 0\\ 0 & F_{1t} & F_{1m} & 0 & F_{2t} & F_{2m} \end{array}\right]v_{p}=Fv_{p}.$$

The shape function matrix has been developed considering a beam of unit length. Hence, scaling for the beam of length, L , we use the dummy variable, $\zeta =x_{1}/L$ . The terms of F are shown in the set of equation (3.16a–f).


(3.16*a*) $$F_{1a}=1-\zeta ,$$

(3.16*b*) $$F_{2a}=\zeta ,$$

(3.16*c*) $$F_{1t}=1-3\zeta^{2}+2\zeta^{3},$$

(3.16*d*) $$F_{1m}=L\left(\zeta -2\zeta^{2}+\zeta^{3}\right),$$

(3.16*e*) $$F_{2t}=3\zeta^{2}-2\zeta^{3},$$

(3.16*f*) $$F_{2m}=L\left(-\zeta^{2}+\zeta^{3}\right).$$

Separating the neutral axis deformation terms, we have equation (3.17*a,* *b)*.


(3.17*a*) $$v_{1}^{c}=\left[1\;\;\;0\right]Fv_{p},$$

(3.17*b*) $$v_{3}^{c}=\left[0\;\;\;1\right]Fv_{p}.$$

Taking variational after differentiating the above expressions, we arrive up to equation (3.18*a,* *b)*. For simplification, we create the matrices, $\bar{N_{\text{1}}}$ and $\bar{N_{\text{2}}}$ .


(3.18*a*) $$\delta v_{1}^{c}{,}_{x_{1}}={\left(\left[1\;\;0\right]F\right)}_{,x_{1}}\delta v={\bar{N}}_{1}\delta v_{p},$$

(3.18*b*) $$\delta v_{3}^{c}{,}_{x_{1}x_{1}}={\left(\left[0\;\;1\right]F\right)}_{,x_{1}x_{1}}\delta v={\bar{N}}_{2}\delta v_{p}.$$

Keeping the voltage, V , as constant, we take the variational of the summation of the total potential energy in equation (3.3) and the internal energy in the piezoelectric layer. Then we get equation (3.19).


(3.19) $$\begin{array}{ll} \delta T_{P} & =\delta v_{p}^{\text{T}}\left(\left(E_{s}A_{s}+E_{p}A_{p}\right)N_{11}-\left(E_{s}I_{s}^{1}+E_{p}I_{p}^{1}\right)N_{12}\right)\delta v_{p}\\ & -\delta v_{p}^{\text{T}}\left(\left(\left(E_{s}I_{s}^{1}+E_{p}I_{p}^{1}\right)N_{21}+\left(E_{s}I_{s}^{2}+E_{p}I_{p}^{2}\right)N_{22}\right)\delta v_{p}+\Phi \right) \end{array}.$$

To simplify equation (3.19), we develop a few constants as shown in the set of equation (3.20a–e). They include the integration terms separated from the material and geometrical constant property terms.


(3.20*a*) $$N_{11}=\int_{\;\;0}^{L}{\bar{N_{\text{1}}}}^{\text{T}}\bar{N_{\text{1}}}dx_{1},$$

(3.20*b*) $$N_{12}=\int_{\;\;0}^{L}{\bar{N_{\text{1}}}}^{\text{T}}\bar{N_{\text{2}}}dx_{1},$$

(3.20*c*) $$N_{12}=\int_{\;\;0}^{L}{\bar{N_{\text{1}}}}^{\text{T}}\bar{N_{\text{2}}}dx_{1},$$

(3.20*d*) $$N_{22}=\int_{\;\;0}^{L}{\bar{N_{\text{2}}}}^{\text{T}}\bar{N_{\text{2}}}dx_{1},$$

(3.20*e*) $$\Phi =\int_{\;\;0}^{L}\frac{1}{2}C_{1}V{\bar{N_{\text{2}}}}^{\text{T}}dx_{1}-\int_{\;\;0}^{L}\frac{1}{2}C_{2}V{\bar{N_{\text{2}}}}^{\text{T}}dx_{1}.$$

Now, we can introduce the stiffness matrix term, $K_{p}$ for the piezoelectric patch, as given by equation (3.21*b)*.


(3.21*a*) $$\delta T_{P}=\delta v_{p}^{\text{T}}\left(K_{p}\delta v_{p}+\Phi \right),$$

(3.21*b*) $$K_{p}=\left(\left(E_{s}A_{s}+E_{p}A_{p}\right)N_{11}-\left(E_{s}I_{s}^{1}+E_{p}I_{p}^{1}\right)N_{12}-\left(E_{s}I_{s}^{1}+E_{p}I_{p}^{1}\right)N_{21}+\left(E_{s}I_{s}^{2}+E_{p}I_{p}^{2}\right)N_{22}\right).$$

The internal energy term can be separately written as


(3.22) $$\delta U_{p}=-\delta v_{p}^{\text{T}}\Phi .$$

Using Hamilton’s principle in equation (3.23), we get the final expression for the equilibrium equation.


(3.23) $$\int_{\;\;t_{1}}^{t_{2}}\left(\delta T_{P}-\delta U_{p}\right)dt=\int_{\;\;t_{1}}^{t_{2}}\delta v_{p}^{\text{T}}\left(K_{p}v_{p}+2\Phi \right)dt=\int_{\;\;t_{1}}^{t_{2}}\delta v_{p}^{\text{T}}\left(L_{p}+2\Phi \right)dt=0.$$

Now, the load vector can be separately represented as shown in equation (3.24*a* *,b*) and it has terms that are linearly dependent upon the voltage value along the piezoelectric patch.


(3.24*a*) $$L_{p}+2\Phi =0\Rightarrow L_{p}=-2\Phi ,$$

(3.24*b*) $$L_{p}^{\text{T}}=\left[\begin{array}{cccccc} C_{1}V & 0 & -C_{2}V & -C_{1}V & 0 & C_{2}V \end{array}\right].$$

Expanding the stiffness matrix, $K_{p}$ , we get the following matrix.


(3.25) $${\text{K}}_{\text{p}}=\left[\begin{array}{cccccc} \frac{E_{s}A_{s}+E_{p}A_{p}}{L} & 0 & -\frac{E_{s}I_{s}^{1}+E_{p}I_{p}^{1}}{L} & -\frac{E_{s}A_{s}+E_{p}A_{p}}{L} & 0 & \frac{E_{s}I_{s}^{1}+E_{p}I_{p}^{1}}{L}\\ 0 & 12\frac{E_{s}I_{s}^{2}+E_{p}I_{p}^{2}}{L^{3}} & 6\frac{E_{s}I_{s}^{2}+E_{p}I_{p}^{2}}{L^{2}} & 0 & -12\frac{E_{s}I_{s}^{2}+E_{p}I_{p}^{2}}{L^{3}} & 6\frac{E_{s}I_{s}^{2}+E_{p}I_{p}^{2}}{L^{2}}\\ -\frac{E_{s}I_{s}^{1}+E_{p}I_{p}^{1}}{L} & 6\frac{E_{s}I_{s}^{2}+E_{p}I_{p}^{2}}{L^{2}} & 4\frac{E_{s}I_{s}^{2}+E_{p}I_{p}^{2}}{L} & \frac{E_{s}I_{s}^{1}+E_{p}I_{p}^{1}}{L} & -6\frac{E_{s}I_{s}^{2}+E_{p}I_{p}^{2}}{L^{2}} & 2\frac{E_{s}I_{s}^{2}+E_{p}I_{p}^{2}}{L}\\ -\frac{E_{s}A_{s}+E_{p}A_{p}}{L} & 0 & \frac{E_{s}I_{s}^{1}+E_{p}I_{p}^{1}}{L} & \frac{E_{s}A_{s}+E_{p}A_{p}}{L} & 0 & \frac{E_{s}I_{s}^{1}+E_{p}I_{p}^{1}}{L}\\ 0 & -12\frac{E_{s}I_{s}^{2}+E_{p}I_{p}^{2}}{L^{3}} & -6\frac{E_{s}I_{s}^{2}+E_{p}I_{p}^{2}}{L^{2}} & 0 & 12\frac{E_{s}I_{s}^{2}+E_{p}I_{p}^{2}}{L^{3}} & -6\frac{E_{s}I_{s}^{2}+E_{p}I_{p}^{2}}{L^{2}}\\ \frac{E_{s}I_{s}^{1}+E_{p}I_{p}^{1}}{L} & 6\frac{E_{s}I_{s}^{2}+E_{p}I_{p}^{2}}{L^{2}} & 2\frac{E_{s}I_{s}^{2}+E_{p}I_{p}^{2}}{L} & -\frac{E_{s}I_{s}^{1}+E_{p}I_{p}^{1}}{L} & -6\frac{E_{s}I_{s}^{2}+E_{p}I_{p}^{2}}{L^{2}} & 4\frac{E_{s}I_{s}^{2}+E_{p}I_{p}^{2}}{L} \end{array}\right].$$

We already know that the mechanical stiffness matrix $K_{m}$ can be given as


(3.26) $${\text{K}}_{\text{m}}=1.5\left[\begin{array}{cccccc} \frac{E_{s}A_{s}+E_{p}A_{p}}{L} & 0 & 0 & -\frac{E_{s}A_{s}+E_{p}A_{p}}{L} & 0 & 0\\ 0 & 12\frac{E_{s}I_{s}^{2}+E_{p}I_{p}^{2}}{L^{3}} & 6\frac{E_{s}I_{s}^{2}+E_{p}I_{p}^{2}}{L^{2}} & 0 & -12\frac{E_{s}I_{s}^{2}+E_{p}I_{p}^{2}}{L^{3}} & 6\frac{E_{s}I_{s}^{2}+E_{p}I_{p}^{2}}{L^{2}}\\ 0 & 6\frac{E_{s}I_{s}^{2}+E_{p}I_{p}^{2}}{L^{2}} & 4\frac{E_{s}I_{s}^{2}+E_{p}I_{p}^{2}}{L} & 0 & -6\frac{E_{s}I_{s}^{2}+E_{p}I_{p}^{2}}{L^{2}} & 2\frac{E_{s}I_{s}^{2}+E_{p}I_{p}^{2}}{L}\\ -\frac{E_{s}A_{s}+E_{p}A_{p}}{L} & 0 & 0 & \frac{E_{s}A_{s}+E_{p}A_{p}}{L} & 0 & 0\\ 0 & -12\frac{E_{s}I_{s}^{2}+E_{p}I_{p}^{2}}{L^{3}} & -6\frac{E_{s}I_{s}^{2}+E_{p}I_{p}^{2}}{L^{2}} & 0 & 12\frac{E_{s}I_{s}^{2}+E_{p}I_{p}^{2}}{L^{3}} & -6\frac{E_{s}I_{s}^{2}+E_{p}I_{p}^{2}}{L^{2}}\\ 0 & 6\frac{E_{s}I_{s}^{2}+E_{p}I_{p}^{2}}{L^{2}} & 2\frac{E_{s}I_{s}^{2}+E_{p}I_{p}^{2}}{L} & 0 & -6\frac{E_{s}I_{s}^{2}+E_{p}I_{p}^{2}}{L^{2}} & 4\frac{E_{s}I_{s}^{2}+E_{p}I_{p}^{2}}{L} \end{array}\right].$$

  We assume a small deformation field in this work. The overall displacement vector v for a single beam can be represented as the summation of two separate displacement vectors that we get from the mechanical and electric loading separately, i.e. $v_{p}$ and $v_{m}$ ,


(3.27) $$v=v_{p}+v_{m}=K_{p}^{-1}L_{p}+K_{m}^{-1}L_{m}.$$

### (b) Voltage-dependent deformation field of piezoelectric lattices

The basic development of a honeycomb lattice considering the composite piezoelectric beams is shown in figure 2. The beam-level stiffness matrices are assembled to obtain the global stiffness matrix of the entire piezoelectric lattice. Subsequently, the voltage-dependent displacement field of the lattice is derived based on applied far-field stresses (resulting in lattice-level nodal load vector) and boundary conditions through adopting appropriate matrix inversion schemes. The global node and element numbering schemes are shown in subfigures (*d*, *f*). The global node numbering is initiated row-wise and we move from the bottom row to the top row, in each row we move from left to right. The order of the element numbering (counter-clockwise) is shown in a hexagonal unit cell for the odd-numbered rows. For the even rows, only the vertical members are marked (the inclined beams are included in the odd rows). The global element numbering is initiated row-wise and we move from the bottom row to the top row, in each row we move from left to right. Assuming a small deformation problem allows us to separately calculate the displacement vectors resulting from the mechanical and piezoelectric loading, and superimpose them to obtain the compound effect of mechanical far-field stress and electrical stimuli. The developed lattice-level code is generic in nature, and capable of accepting the number of cells in two perpendicular directions along with unit-cell-level intrinsic material and geometric parameters as input to obtain the corresponding strain field as output under any given loading and boundary conditions.

### (c) Minimization solver for cloaking

A brief theory regarding *fmincon* solver [57,58] that follows an interior point algorithm [59,60] has been discussed as we adopt it explicitly to achieve the minimization objectives concerning cloaking. Here, we have limited the number of optimization variables to that in the cloaking region (unless otherwise mentioned), leading to a reduced dependency of the displacement norm to the minimum number of degrees of freedom. We have adopted this strategy to solve the problem with better storage efficiency and minimum time. As it would be evident in the following section that we are able to achieve the target modified deformation field of the damaged lattice through an automated voltage augmentation factor.

For a particular problem, whether it is linear or nonlinear, convex or non-convex, interior point approaches can be applied. Both huge, sparse constraints and small, dense problems can be handled by interior point algorithms. The algorithm can recover from undefined outcomes like complex and infinity solutions and fulfils limits at all iterations. In the current work, we have used *fmincon*, a nonlinear programming solver in Matlab [61]. It uses an interior point algorithm as a default tool to solve optimization problems (refer to equation (3.28)). To find the restricted minimum of the nonlinear multi-variable function, *fmincon* is generally used as an optimization solver. It uses sequential quadratic programming, trust areas, and a barrier method considering slack variables (refer to equations (3.29) and (3.30)) to tackle the subproblems that arise throughout the iteration. First, an ideal subproblem is put up in the sequential quadratic programming model to look for the following feasible point in the present iteration point. Trust regions in the domain of the problem help in converging the problem. A barrier function in constrained optimization is a continuous function whose value on a point grows to infinity as the point gets closer to the limit of the viable region of an optimization problem. Such functions are employed, substituting an easier way to manage penalized terms in the goal function for inequality restrictions. Generally, *fmincon* uses a logarithmic barrier function, that impacts the objective function by considering a gradient over the values closer and away from the extreme values. When the objective function is non-decreasing in practical directions, to within the value of the optimality tolerance, and constraints are met, to within the value of the constraint tolerance, it indicates that the optimization is accomplished.


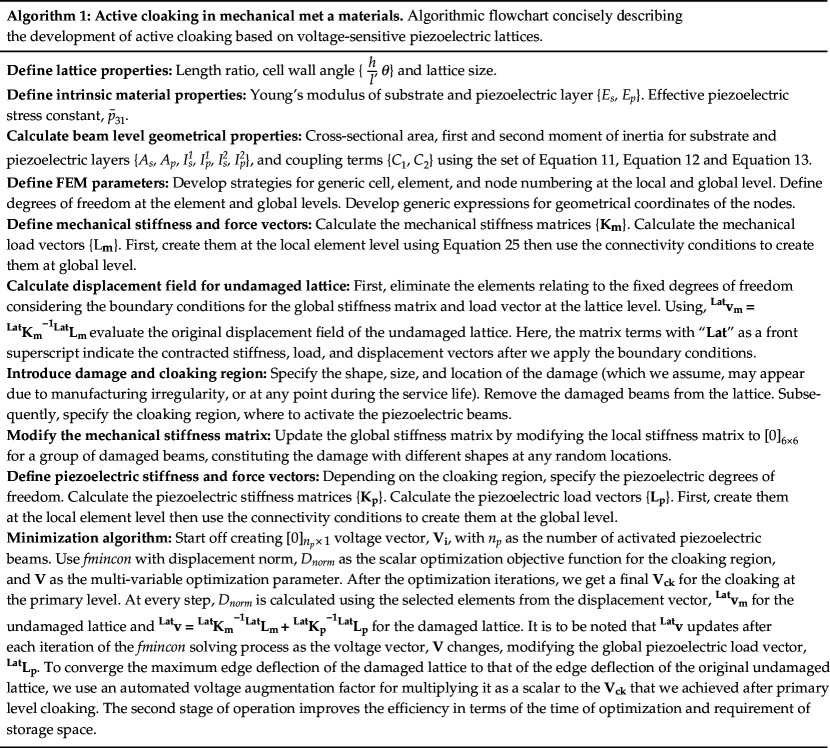



(3.28) $$\min\;\;opt\left(\bar{v}\right)\;\;\text{such}\;\text{that}\;\;\left[\begin{array}{c} nc\left(\bar{v}\right)\leq 0\\ nc_{eqv}\left(\bar{v}\right)=0\\ A.\bar{v}\leq B\\ A_{eqv}.\bar{v}\leq B_{eqv}\\ lw_{b}\leq \bar{v}\leq up_{b} \end{array}\right].$$

In the above set of equation (3.28), $nc(\bar{v})$ and $nc_{eqv}(\bar{v})$ represent the set of nonlinear inequality and equality constraints through arrays. In a similar manner, A , B and $A_{eqv}$ , $B_{eqv}$ represent the set of linear inequality and equality constraints, respectively. Here, $lw_{b}$ and $up_{b}$ are the lower and upper bounds for the minimization problem.


(3.29) $$\min_{\bar{v}}\;\;opt\left(\bar{v}\right)\;\;\text{subject to}\;\;g_{i}^{1}\left(\bar{v}\right)=0\;\;\text{and}\;\;g_{i}^{2}\left(\bar{v}\right)\leq 0,$$

(3.30) $$\min_{\bar{v},s^{l}}\;\;opt_{\gamma ,s^{l}}\left(\bar{v}\right)=\min_{\bar{v}}-\gamma \Sigma ln\left(s_{i}^{l}\right)\;\;\text{subject to}\;\;s^{l}\geq 0,\;\;g_{i}^{1}\left(\bar{v}\right)=0\;\;\text{and}\;\;g_{i}^{2}\left(\bar{v}\right)+s^{l}=0.$$

The number of slack variables, $s_{i}^{l}$ is equal to that of the number of inequality constraints, $g_{i}^{2}$ . Now, the slack variables have to be positive to keep the iterations running in the feasible domain. For each of γ>0 , we have equation (3.30).


(3.31) $$L\left(\bar{v},\lambda \right)=opt\left(\bar{v}\right)+\Sigma \lambda_{g_{i}^{1}}g_{i}^{1}\left(\bar{v}\right)+\Sigma \lambda_{g_{i}^{2}}g_{i}^{2}\left(\bar{v}\right),$$

(3.32) $$\nabla_{,\bar{v}\bar{v}}^{2}L\left(\bar{v},\lambda \right)=\nabla^{2}opt\left(\bar{v}\right)+\Sigma \lambda_{g_{i}^{1}}\nabla^{2}g_{i}^{1}\left(\bar{v}\right)+\Sigma \lambda_{g_{i}^{2}}\nabla^{2}g_{i}^{2}\left(\bar{v}\right).$$

*Fmincon* solver uses finite differences to estimate gradients if derivatives are not provided by the user. While a finite-difference estimate may be quicker for difficult derivatives, if we supply gradients, the solver need not conduct this finite-difference estimation, allowing it to run faster and be more accurate. Additionally, solvers employ an approximation of the Hessian, which might differ greatly from the actual Hessian. A Hessian can speed up the process of finding a solution. The Hessian of the Lagrangian in *fmincon* algorithm is represented by equations (3.31) and (3.32). Here, the terms $\lambda_{g_{i}^{1}}$ and $\lambda_{g_{i}^{2}}$ represent the Lagrangian multipliers. The *fmincon* solver generally has different algorithms with diverse Hessian options.

#### (i) Definition of displacement norm and cloaking region

For the current optimization problem performed in this paper, we have tried to minimize $D_{norm}$ , which is given by equation (3.33). Here $n_{p}$ stands for the degree of freedoms (all including axial, transverse and rotational) that is included in the cloaking region constituting the activated piezoelectric beams. Now, it is to be noted that we have used a scalar objective function, but the optimization problem has multiple design variables, i.e. the different voltage values of the activated beams stored in V . The design variables are related to the global displacement vector of the lattice, nonlinearly through the finite element formulation.


(3.33) $$D_{norm}=\sum_{i=1}^{n_{p}}{\left(\;\text{Lat}v_{m}\left(i\right){-}^{\text{Lat}}v\left(i\right)\right)}^{2}$$

The constraint is provided here in terms of the range of applied voltage. In the above equation for $D_{norm}$ , $\;\text{Lat}v_{m}$ and  Latv represent the displacements of undamaged lattices without any applied voltage and the displacements of damaged lattices with externally applied voltage, respectively. This leads to a cloaking effect with minimal difference in the displacement fields of undamaged and damaged lattices beyond the cloaking region.

It can be noted that the entire lattice is made of piezoelectric components here but the members are electrically activated selectively based on the respective value of supplied voltage. Depending on the location and nature of the damage, the cloaking region can be defined in an on-demand framework by supplying necessary electrical stimuli to the members within that region only, leading to an unprecedented active and on-demand cloaking. We have chosen the cloaking region to be around the damage (unless otherwise mentioned) instead of activating (i.e. applying voltage) all the undamaged beams. This results in a reduction of the dependence of the norm, $D_{norm}$ on more degrees of freedom, instead, we only use the degrees of freedom inside the cloaking region. It also reduces the optimization time and energy requirement and does not affect our results. The shape of the cloaking region is determined based on an algorithmic understanding coupled with intuition. For example, as it would be evident in the results section, while considering unidirectional loading along direction 1 and for shear loading along direction 12, we have shifted the cloaking region along the loading direction. This has been adopted to increase the cloaking effect by providing more space in the highly affected regions to negate the effect of altered strain fields owing to damage. Our final aim is to converge the deformation field beyond the cloaking region (and near the loading edge) of the damaged lattice to that of the edge deformation in an undamaged lattice. We achieve the final cloaking using an automated voltage augmentation factor to modify the voltage vector resulting from the *fmincon* solver, leading to reduction in the time of optimization and requirement of storage space.

In this context, it is worth mentioning that the number of design variables (i.e. number of electrically active beam members having non-zero voltage) in the minimization algorithm increases significantly as the area of the cloaking region increases, leading to an escalation in the computational expenses. However, a bigger cloaking region may provide better cloaking effect beyond this region. Thus, there exists a trade-off between these two aspects, and we have tried to minimize the cloaking area through an automated and iterative algorithm in the current study. In case of multiple damages, if they are too scattered as an extreme case, the cloaking region can be considered throughout the lattice (except for the edge rows), as depicted later in figure 8.

#### (ii) Stopping criteria of the minimization solver

It is important to precisely define when the iterations in the optimization algorithm stop, which depends on different threshold values and tolerances. The adopted minimization solver allows the user to set different tolerance values including the number of iterations, optimality measure, constraints, norm value, etc. For the *fmincon* solver, the tolerance values are generally relative, i.e. scaling is done with respect to the problem size. We should make sure that the optimization curve has converged. Depending on the nonlinear optimization problem, the objective function may not minimize as we expect. Also, results are not always precise when very small tolerances are set. Instead, a solution might not be able to tell when it has converged, leading to pointless repetitions. *Fmincon* iterations will generally stop when any of the function values may surpass the evaluation limit or when the objective function plot converges and stops reducing.

## 4. Results and discussion

The design parameters for the lattice construction are briefly discussed first, followed by boundary and loading conditions. In this context, it may be noted that the proposed methodology for active cloaking is generic in nature and it can be implemented in any other lattice forms. Before presenting the results concerning active cloaking, we have adopted a multi-stage validation approach involving the voltage-dependent response of the unimorph beam elements, lattice-level effective mechanical properties without damage and effective electromechanical properties of metamaterials. The results concerning such validation with the literature [22,38,62,63] are presented in electronic supplementary material, figure S1. Subsequently, we would demonstrate in the following subsection the concept of active cloaking in lattice-based mechanical materials considering a range of defect shapes and sizes, along with their numbers, random locations and multiple scenarios of normal, shear and compound mixed-mode far-field externally applied stresses.

### (a) Lattice- and beam-level geometrical and material properties

In the present work, we have considered honeycomb lattices having composite beams of width, B as $200\times 10^{-3}$ mm with $100\times 10^{-3}$ -mm thick ( $H_{s}$ ) substrate of Young’s modulus, $E_{s}$ as 70 GPa, and a $50\times 10^{-3}$ -mm thick ( $H_{p}$ ) piezoelectric layer of Young’s modulus, $E_{p}$ as 127 GPa, bonded above the substrate. The piezoelectric layer has an effective piezoelectric constant, $p_{31}^{k}$ of $-2.74\times 10^{-10}$ m/V. The size of the honeycomb lattice is taken as 25×25 with a cell wall angle of $50^{{}^{\circ}}$ for demonstrating the proposed concepts numerically. The length of the inclined members is considered as 20 mm and that of the vertical members as 30 mm.

### (b) Loading and boundary conditions

As discussed in the earlier sections, the main objective of the current work is to hide (or minimize) the additional deformation perceived by a hexagonal lattice when subjected to different types of loading. To start off, we try uni-modal axial loading (or in other words, far-field applied external stresses to the honeycomb lattice) along direction 1, direction 2 and shear loading along direction 12. Subsequently, more complex mixed-mode loading scenarios are considered. Schematic representations of all the uni-modal or mixed-mode loading scenarios are furnished in figure 2*g–k*.

For axial loading on the lattice along direction 1, the degree of freedom along direction 1 of the nodes spanning the left edge of the lattice is fixed and the loading is applied to the nodes of the opposite edge along the same direction. For axial loading on the lattice along direction 2, the degree of freedoms along direction 2 of the nodes spanning the bottom edge of the lattice is fixed and the loading is applied to the nodes of the top edge along the same direction. For shear loading on the lattice along direction 12, both the degrees of freedom along directions 1 and 2 of the nodes spanning the bottom edge of the lattice are fixed, and the loading is applied to the nodes of the top edge along direction 1. The loading and boundary conditions for mixed-mode loading scenarios are obtained by superimposing the cases of normal and shear modes, as depicted in figure 2*j,k*. To consider the stability of the lattice system (such that it does not displace as a rigid body), we fix both the translational degrees of freedom of a corner node in all the loading scenarios. For all the loading cases that we have considered, either uni-modal or mixed-mode, we have applied a stress magnitude of 25 Pa.

### (c) Displacement field representation

**Figure 3.**
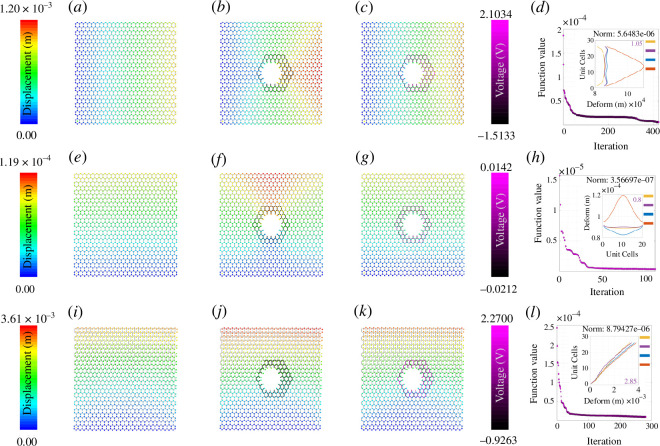
Active mechanical cloaking of a lattice with single hexagonal damage at the centre under uni-modal loading. (*a, e, i*) Deformation field of a lattice metamaterial without damage subjected to axial loading along directions 1 and 2, and shear loading along direction 12. (*b, f, j*) Deformation field of the same lattice metamaterial with damage subjected to axial loading along directions 1 and 2, and shear loading along direction 12. The black thickened beams around the damage represent the cloaking region. (*c, g, k*) Deformation field of the same lattice metamaterial with damage, subjected to axial loading along directions 1 and 2, and shear loading along direction 12 (under active piezoelectric cloaking). The thickened beams around the damage with shades of purple represent the electrically activated cloaking region with different voltage values supplied to the beams (note the purple colour bars). (*d, h, l*) Iteration and convergence of the minimization algorithm along with deformation of the loaded edges for different cases (yellow: undamaged case, orange: damaged case without cloaking, blue: cloaking with exact *fmincon* voltage output, purple: cloaking with the voltage output of the bi-level minimization algorithm involving *fmincon* and automated voltage augmentation factor).

**Figure 4.**
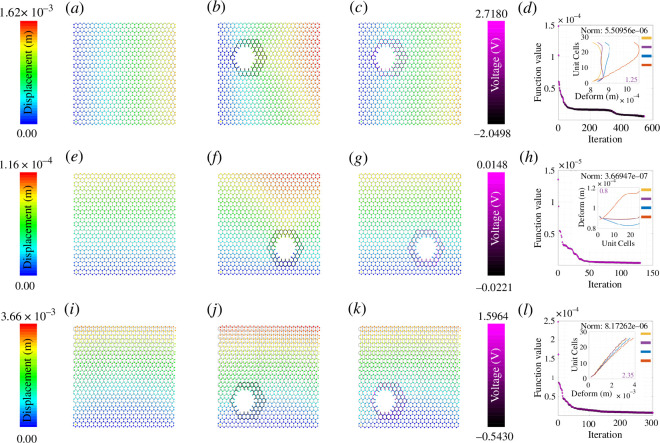
Active mechanical cloaking of a lattice with a single hexagonal damage at any random position under uni-modal loading. (*a, e, i*) Deformation field of a lattice metamaterial without damage subjected to axial loading along directions 1 and 2, and shear loading along direction 12. (*b, f, j*) Deformation field of the same lattice metamaterial with damage subjected to axial loading along directions 1 and 2, and shear loading along direction 12. The black thickened beams around the damage represent the cloaking region. (*c, g, k*) Deformation field of the same lattice metamaterial with damage, subjected to axial loading along directions 1 and 2, and shear loading along direction 12 (under active piezoelectric cloaking). The thickened beams around the damage with shades of purple represent the electrically activated cloaking region with different voltage values supplied to the beams (note the purple colour bars). (*d, h, l*) Iteration and convergence of the minimization algorithm along with deformation of the loaded edges for different cases (yellow: undamaged case, orange: damaged case without cloaking, blue: cloaking with exact *fmincon* voltage output, purple: cloaking with the voltage output of the bi-level minimization algorithm involving *fmincon* and automated voltage augmentation factor).

**Figure 5.**
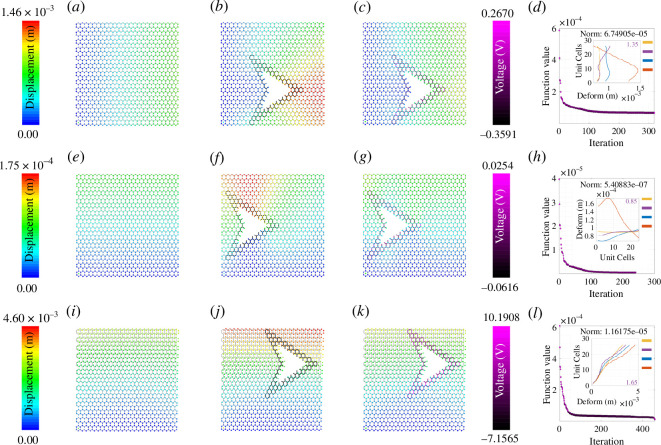
Active mechanical cloaking of a lattice with a single arrow-shaped damage at any random position under uni-modal loading. (*a, e, i*) Deformation field of a lattice metamaterial without damage subjected to axial loading along directions 1 and 2, and shear loading along direction 12. (*b, f, j*) Deformation field of the same lattice metamaterial with damage subjected to axial loading along directions 1 and 2, and shear loading along direction 12. The black thickened beams around the damage represent the cloaking region. (*c, g, k*) Deformation field of the same lattice metamaterial with damage, subjected to axial loading along directions 1 and 2, and shear loading along direction 12 (under active piezoelectric cloaking). The thickened beams around the damage with shades of purple represent the electrically activated cloaking region with different voltage values supplied to the beams (note the purple colour bars). (*d, h, l*) Iteration and convergence of the minimization algorithm along with deformation of the loaded edges for different cases (yellow: undamaged case, orange: damaged case without cloaking, blue: cloaking with exact *fmincon* voltage output, purple: cloaking with the voltage output of the bi-level minimization algorithm involving *fmincon* and automated voltage augmentation factor).

**Figure 6.**
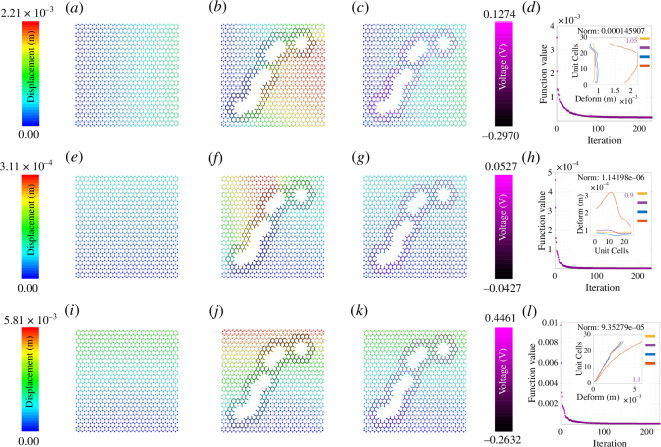
Active mechanical cloaking of a lattice with a random damage shape at any random position under uni-modal loading. (*a, e, i*) Deformation field of a lattice metamaterial without damage subjected to axial loading along directions 1 and 2, and shear loading along direction 12. (*b, f, j*) Deformation field of the same lattice metamaterial with damage subjected to axial loading along directions 1 and 2, and shear loading along direction 12. The black thickened beams around the damage represent the cloaking region. In this case, the damage configuration is developed using the overlap of multiple hexagonal shapes. (*c, g, k*) Deformation field of the same lattice metamaterial with damage, subjected to axial loading along directions 1 and 2, and shear loading along direction 12 (under active piezoelectric cloaking). The thickened beams around the damage with shades of purple represent the electrically activated cloaking region with different voltage values supplied to the beams (note the purple colour bars). The cloaking region is formed with the union of cloaking regions corresponding to each of the damaged shapes. (*d, h, l*) Iteration and convergence of the minimization algorithm along with deformation of the loaded edges for different cases (yellow: undamaged case, orange: damaged case without cloaking, blue: cloaking with exact *fmincon* voltage output, purple: cloaking with the voltage output of the bi-level minimization algorithm involving *fmincon* and automated voltage augmentation factor).

**Figure 7.**
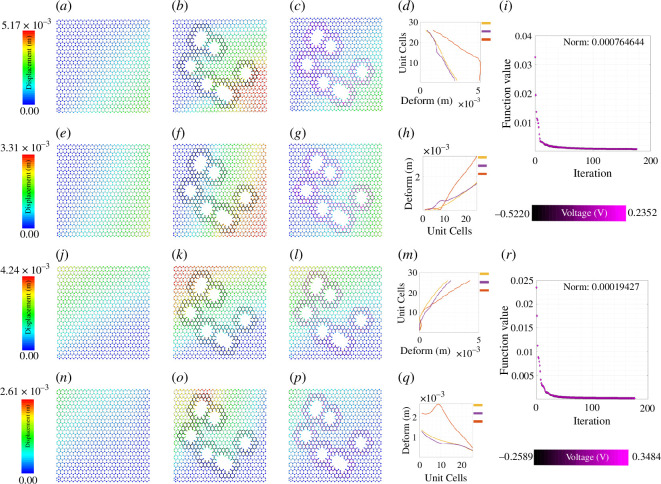
Active mechanical cloaking of a lattice with a random damage shape at any number of random positions under mixed-mode loading. (*a, e*) Deformation field of a lattice metamaterial without damage subjected to axial loading along direction 1 along with shear loading along direction 21. (*j, n*) Deformation field of a lattice metamaterial without damage subjected to axial loading along direction 2 along with shear loading along direction 12. (*b, k, c, l*) Deformation gradient along direction 1 for the uncloaked and cloaked lattices with damage. (*f, o, g, p*) Deformation gradient along direction 2 for the uncloaked and cloaked lattices with damage. Note that the column of subfigures (*a, e, j, n*) represents the deformation field of undamaged lattices, while the column of subfigures (*b, f, k, o*) and (*c, g, l, p*) represent damaged uncloaked and damaged cloaked lattices, respectively. The black thickened beams around the damage represent the cloaking region. In this case, the damage configuration is developed using the overlap of multiple hexagonal shapes. The thickened beams around the damage with shades of purple represent the electrically activated cloaking region with different voltage values supplied to the beams (note the purple colour bars). The cloaking region is formed with the union of cloaking regions corresponding to each of the damaged shapes. (*d, m, h, q*) Edge deformation for the right and top sides of the lattice. (*i, r*) Iteration and convergence of the minimization algorithm along with deformation of the loaded edges for different cases (yellow: undamaged case, orange: damaged case without cloaking, blue: cloaking with exact *fmincon* voltage output, purple: cloaking with the voltage output of the bi-level minimization algorithm involving *fmincon* and automated voltage augmentation factor).

**Figure 8.**
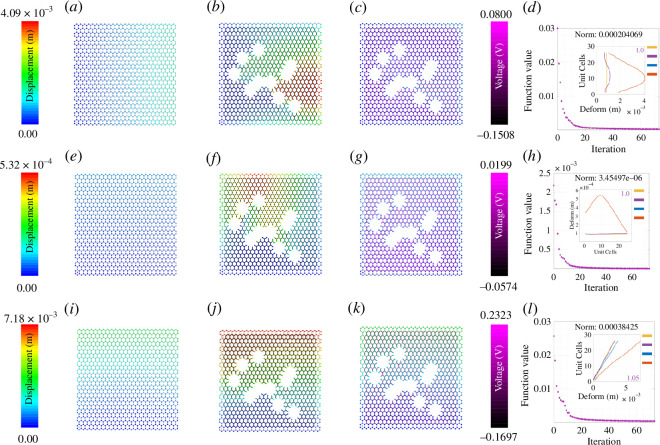
Active mechanical cloaking of a lattice with multiple randomly shaped damages at any random position scattered around a large part of the lattice domain under uni-modal loading. (*a, e, i*) Deformation field of a lattice metamaterial without damage subjected to axial loading along directions 1 and 2, and shear loading along direction 12. (*b, f, j*) Deformation field of the same lattice metamaterial with damage subjected to axial loading along directions 1 and 2, and shear loading along direction 12. The black thickened beams around the damage represent the cloaking region. In this case, the damage configuration is developed using the overlap of multiple hexagonal shapes. (*c, g, k*) Deformation field of the same lattice metamaterial with damage, subjected to axial loading along directions 1 and 2, and shear loading along direction 12 (under active piezoelectric cloaking). The thickened beams around the damage with shades of purple represent the electrically activated cloaking region with different voltage values supplied to the beams (note the purple colour bars). The cloaking region is formed with the union of cloaking regions corresponding to each of the damaged shapes. (*d, h, l*) Iteration and convergence of the minimization algorithm along with deformation of the loaded edges for different cases (yellow: undamaged case, orange: damaged case without cloaking, blue: cloaking with exact *fmincon* voltage output, purple: cloaking with the voltage output of the bi-level minimization algorithm involving *fmincon* and automated voltage augmentation factor).

The colour gradient ranging from dark blue to red indicates deformation ranging from zero to the maximum value. It is clear from figures 3–8 that when the damage is introduced without activating the piezopatches, the deformation increases which is indicated by the red region. Later, different activating voltage values on the piezopatches allow the reduction of the deformation close to the original undamaged lattice. For a better understanding, the edge deformation of the loaded edges is shown separately. We have used four coloured legends for all these figures, yellow for the undamaged case, orange for the damaged case without cloaking and blue for the cloaking case when the exact *fmincon* voltage values are used. Finally, we have shown in purple colour the edge deformation when an automated voltage augmentation factor is multiplied by the *fmincon* voltage matrix. This factor allows us to converge the final edge deformation to the edge deformation of the undamaged case, to the extent that the maximum amount of the additional edge deformation has been reduced. Even though the optimization problem is nonlinear, *fmincon* manages to give a voltage matrix that when operated linearly with the automated voltage augmentation factor leads to the additional edge deformation reduction. We have seen earlier in §3 that the piezoelectric load vector in equation (3.24*a* *,b*) is linearly dependent upon the voltage of the beam. Now considering the overall lattice with the cloaking region with different beam voltages, the automated voltage augmentation factor allows voltage enhancement of each of the beams such that the global deformation reduces.

It is to be noted that, for the case of uni-modal and mix-modal loading (i.e. normal far-field loading in direction 1, direction 2, or far-field shear loading), the deformed nodal positions indicate the final position of the disturbed nodes. However, the colour gradient in case of uni-modal loading is shown depending explicitly on the loading direction, i.e. for loading along direction 1, the colour gradient ranges only for the direction 1 deformation of the nodes. Similarly, for loading along direction 2, the colour gradient ranges only for the direction 2 deformation of the nodes. For shear loading along direction 12, the colour gradient ranges only for the direction 1 deformation of the nodes. For the mixed-mode loading cases, as there is not a single direction of externally applied stress similar to the uni-modal case, we present the colour gradient considering the values of normal displacements both in directions 1 and 2 for each of the nodes. A similar colour bar with shades of purple has been shown to properly depict the voltage values after we activate the piezopatches. It can be noted that the presented voltage values are for the final case, when we use the automated voltage augmentation factor to converge the edge deformation to the maximum possible extent.

### (d) Optimization iterations for different damage shapes

The subfigures (*d*, *h*, *l*) in figures 3–8 show the optimization iterations indicating the norm reduction as the simulation progresses. For most of the cases, the solver settings allow us to get the final optimized voltage values on the beams iterating below 600 times. It has been found that bi-symmetrical damage shapes like a hexagon (figure 3) and star (electronic supplementary material, figure S3) give better optimization to both the axial loading types. Damage shapes that are symmetrical only along one axis, for example triangle (electronic supplementary material, figure S4) and arrow shapes (electronic supplementary material, figure S5) give relatively better deformation reduction when the loading direction is parallel to the direction of the damage symmetry. Non-symmetrical shapes like the rhombic (electronic supplementary material, figure S6) in this case do not show any specific trends as compared with the other ones. Nevertheless, using *fmincon*, we are able to achieve a satisfactory reduction in the maximum deformation for the central damage location for the cases we have considered. In general, considering the subfigures (*a*, *e*, *i*), the mechanical properties and the configuration of the lattice are such that the overall moduli of the undamaged lattice are higher along direction 2 than that along directions 1 and 12. We can infer about this considering the maximum edge deformation values that are the least for the case of direction 2 loading. It can be seen that the optimization gives lower voltage values for direction 2 loading and it is because of the higher lattice moduli along that direction. On the other hand, the voltage values are higher in case of damage when introduced for the shear loading case owing to the lower overall shear moduli of the hexagonal lattice that we have considered.

### (e) Discussion on progressive complexity in damage scenarios

In this section, we demonstrate active cloaking considering progressively complicated damage scenarios and boundary conditions. In figures 3–8 and electronic supplementary material, figures S2–S14, the subfigures (*a*, *e*, *i*) show the displacement fields of undamaged lattices, subfigures (*b*, *f*, *j*) show the displacement fields of damaged lattices without any external electrical stimuli and subfigures (*c*, *g*, *k*) show the displacement fields of damaged lattices with activated piezoelectric elements (i.e. when external voltage is applied for cloaking). The objective here is to minimize the difference between the deformation fields beyond the cloaking region, and near the loading edges for the undamaged metamaterial (without any applied voltage) and damaged metamaterial (with applied voltage). Thus, if the displacement fields beyond the cloaking region and near the loading edges, presented in subfigures (*a*, *e*, *i*) and (*c*, *g*, *k*), respectively, agree closely, the cloaking algorithm would be treated to have worked satisfactorily. In addition, the insets in subfigures (*d*, *h*, *l*), primarily the curves representing the loading edge deformations of undamaged lattice (yellow) and damage lattice with bi-level optimized voltage input (purple), also ascertains the accuracy of the cloaking algorithm. In lattice metamaterials, having the loading edge deformations unaltered in damaged lattices would mean that the critical properties like effective stiffness or effective Young’s moduli remain unchanged as a result of the mechanical cloaking. In doing so, we need to find out a set of voltage values applied in the piezoelectric members (obtained through the bi-level automated minimization algorithm discussed in the preceding sections) so that such mechanical cloaking can be achieved. It can be noted in this context that the entire lattice is made of piezoelectric components here, but the members are electrically activated selectively based on the respective value of supplied voltage. Depending on the location and nature of damage, the cloaking region can be defined here in an on-demand framework by supplying necessary electrical stimuli to the members within that region only, leading to an unprecedented active and on-demand cloaking.

First, we have investigated single damage at the centre covering different shapes like hexagonal-, rhombic-, triangular-, star- and arrow-shaped damages as shown in figure 3, electronic supplementary material, figures S2–S6 (under unidirectional normal and shear loading). In electronic supplementary material, figure S2, we have increased the damage size as compared with that in figure 3 to check whether our cloaking algorithm works. The second set of figures is for uni-modal axial and shear loading but with non-central damage position as shown in figures 5 and 6 and electronic supplementary material, figures S7–S9. In the previous set of studies with centrally located damage, the edge deformation is likely to be symmetrical before we activate the piezopatches. When we change the position of the damage to any random position, the edge deformation is not symmetrical and the position of the maximum deformation also changes. Hence, the optimization does not give symmetric results as well, similar to that of the central damage with a rhombic shape. However, there is a satisfactory reduction in the deformation value when the piezopatches are activated with the voltage values as given by *fmincon*. Considering the cases for the shear loading for the non-central loading shapes, the edge deformation patterns are similar to those of the central loading shapes. It can be observed that the *fmincon* solver iterates the least before convergence for the uni-modal loading in direction 2 both for central and non-central single loading types. The value of automated voltage augmentation factor is less than one for uni-modal loading in direction 2, and it is the highest for the shear loading in direction 12.

In the next stage, we consider more complicated damage scenarios concerning random damage over the lattice domain as shown in figure 6, electronic supplementary material, figures S10 and S11. Here, we have created random damage patterns considering the overlap of the existing generalized damage shapes. Separate arrays storing the damaged beams and corresponding activated piezoelectric beams are created for each generalized damage shape. Now, let us consider some $r_{d}$ number of that similar shape distributed randomly over the lattice. Hence, we have $r_{d}$ arrays, each for the damaged and cloaking cells. In mathematical terms, we take their union separately which removes the repeating cell numbers. The presented results ascertain that the cloaking algorithm works satisfactorily, albeit the number of optimization iterations to converge depends on the complexity of random damage scenarios.

So far, we have discussed the cloaking under uni-modal far-field applied normal or shear stresses, as depicted in figure 2*g–i*. In the next stage, we consider mixed-mode far-field loading scenarios (refer to figure 2*j,k*). In the first case, we apply an axial load along direction 1, along with a shear loading along direction 12. Here, we resist the transnational degree of freedoms (directions 1 and 2) of the left edge. In the second case, we apply an axial load along direction 2, along with a shear loading along direction 12. Here, we resist the transnational degrees of freedom (directions 1 and 2) of the bottom edge. Another important point to be taken care of is the magnitude of the stress we apply. Earlier, we mentioned that 25 Pa of stress was used for both the uni-modal, axial and shear loading cases. Here, for the first mixed-mode loading, 25 Pa has been used for the direction 1 load and 25 Pa for the direction 21 load. However, for the second mixed-mode loading, 250 Pa has been used for the direction 2 load and 25 Pa for the direction 12 load, to investigate the accuracy of the cloaking algorithm under higher far-field applied stress. The results presented in figure 7, electronic supplementary material, figures S12 and S13 (displacement fields along with the edge deformations for the right and top edges) establish the accuracy of the proposed active cloaking algorithm under random multiple damage scenarios and mixed-mode far-field applied stresses.

Though we have tried to restrict the area of cloaking region thus far keeping the computational expenses in mind, it may sometimes be necessary to consider almost the entire lattice as cloaking region in certain extreme cases of dense random damage distribution spread throughout the entire domain. Notably, the necessary area of cloaking region can be determined by the intelligent metamaterial system through an automated iterative algorithm. Thus, even in such extreme cases, active mechanical cloaking can be achieved in an unsupervised framework. Such extreme damage scenarios are shown in figure 8 (under uni-modal loading) and electronic supplementary material, figure S14 (under mixed-mode loading), where the cloaking region is considered to be the entire lattice except the outer edges. The comparative displacement fields near the edges for undamaged and cloaked damaged lattices show a satisfactory degree of accuracy. In this context, it may be noted that we have not considered failure of individual beam-like members under the compound effect of mechanical and electrical loading in the simulation. Such aspects should be included further while designing active mechanical cloaking for future practical implementations. Consideration of a wider cloaking region would reduce the possibility of element-level failure owing to the higher scope of stress re-distribution for negating the effect of damage.

## 5. Conclusions and perspective

This paper proposes a novel concept of active mechanical cloaking for unsupervised damage shielding in elastic metamaterials. A radically different approach by introducing piezoelectric lattices is developed with element-wise multi-physically controlled cloaks by voltage-dependent modulation of the stress and strain fields. Based on the nature and location of damage(s), the cloaking region can be determined by the intelligent metamaterial system along with the necessary external voltage demand in each of the beam-like members within the cloaking region through an automated algorithm. We envisage in a physical system that the strain measurements can be sent as an output to an external circuit board, which would decide the cloaking region and the inbuilt algorithm would decide the supply voltage to be sent as an input to the lattice structure in an unsupervised framework. Subject to a wide range of uni-modal or mixed-mode loading and boundary conditions, single or multiple disorders and damages with complex shapes, sizes, distribution and numbers can effectively be kept hidden, ensuring extended lifetime and high reliability in the homogenized mechanical behaviour of mechanical metamaterials.

The proposed computational framework for active cloaking in mechanical metamaterials involves a seamless integration of three components: (i) piezoelectric beam elements and their voltage-dependent deformation mechanics, (ii) lattice-level mechanics to obtain the strain field under a given loading and boundary conditions, and (iii) multi-objective discrepancy minimization algorithm between the strain fields of damaged and undamaged lattices. We have adopted a multi-stage validation approach involving the voltage-dependent response of the unimorph beam elements, lattice-level effective mechanical properties without damage and effective electromechanical properties of metamaterials. Subsequently, a detailed numerical investigation is presented to demonstrate the proposed active mechanical cloaking algorithm considering the progressive level of complexity in damage scenarios.

By re-distributing stress and strain components in an active and on-demand framework based on the location, nature, size and number of damages, mechanical cloaking as proposed here will facilitate in hiding essential voids and cut-outs in structural designs, or unintentional defects (either manufacturing or operational in nature) which are often the sources of deviation from intended structural performance and further failure initiation. With improved damage resilience, it will significantly enhance the durability and lifetime of a structure without compromising on mechanical performances like strength and stiffness. Furthermore, since the current framework relies on external multi-physical stimuli like voltage in the cloaking region for altering the stress and strain fields, it is not necessary to reinforce the cloaking region through optimally introduced additional elements in the lattice, as commonly followed in the mechanical cloaks proposed in literature. From a sustainability viewpoint, mechanical cloaking without any additional weight would be crucial for a range of lightweight engineering applications. Since the periodicity in lattice geometry is not broken in the present framework and no additional material is added in the cloaking region, it will be crucial for efficient manufacturing and achieving scalability in production.

Owing to the architected void-filled low-density configurations of metamaterials, they are prone to defects during the complex manufacturing process, or damages under operational conditions. The major drawback in the studies concerning mechanical cloaks so far to negate the effect of damages is that the damage location should be known *a priori*, and the cloak is designed around that damaged zone before manufacturing. Such postulation does not allow unsupervised damage shielding during the manufacturing or service life of the metamaterials by active reconfiguration of the stress fields depending on random and unpredictable evolution of damage. The realization of active cloaking will bring a step-change in the field of mechanical cloaking since the location and intensity of damage (/disorder) do not necessarily need to be known *a priori* before manufacturing. The current elastostatic cloaking algorithm can be extended to the dynamic regime to protect lattices and metamaterials from incident elastic waves by controlling the stiffness locally in a programmable framework. The proposed active cloaking models will further lead to advanced digital twins, wherein damages in intelligent mechanical, aerospace and biomedical structures can be identified in real-time and subsequently the effect of such damages can be eliminated for an uninterrupted mechanical performance.

## Data Availability

All data sets used to generate the results are available in the main paper. Further details could be obtained from the corresponding authors upon reasonable request. Supplementary material is available online [64].
